# Flood Hazard Assessment and Mapping: A Case Study from Australia’s Hawkesbury-Nepean Catchment

**DOI:** 10.3390/s22166251

**Published:** 2022-08-19

**Authors:** Matthew Kelly, Yuriy Kuleshov

**Affiliations:** 1 Bureau of Meteorology, Docklands, VIC 3008, Australia; 2 Science Advanced-Global Challenges Program, Monash University, Clayton, VIC 3800, Australia; 3 SPACE Research Centre, School of Science, Royal Melbourne Institute of Technology (RMIT) University, Melbourne, VIC 3000, Australia

**Keywords:** flood, flood hazard assessment and mapping, flood hazard index, Hawkesbury-Nepean catchment, Australia, flood risk assessments

## Abstract

Floods are among the costliest natural hazards, in Australia and globally. In this study, we used an indicator-based method to assess flood hazard risk in Australia’s Hawkesbury-Nepean catchment (HNC). Australian flood risk assessments are typically spatially constrained through the common use of resource-intensive flood modelling. The large spatial scale of this study area is the primary element of novelty in this research. The indicators of maximum 3-day precipitation (M3DP), distance to river—elevation weighted (DREW), and soil moisture (SM) were used to create the final Flood Hazard Index (FHI). The 17–26 March 2021 flood event in the HNC was used as a case study. It was found that almost 85% of the HNC was classified by the FHI at ‘severe’ or ‘extreme’ level, illustrating the extremity of the studied event. The urbanised floodplain area in the central-east of the HNC had the highest FHI values. Conversely, regions along the western border of the catchment had the lowest flood hazard risk. The DREW indicator strongly correlated with the FHI. The M3DP indicator displayed strong trends of extreme rainfall totals increasing towards the eastern catchment border. The SM indicator was highly variable, but featured extreme values in conservation areas of the HNC. This study introduces a method of large-scale proxy flood hazard assessment that is novel in an Australian context. A proof-of-concept methodology of flood hazard assessment developed for the HNC is replicable and could be applied to other flood-prone areas elsewhere.

## 1. Introduction

### 1.1. Floods

Defined as “an overflow of water beyond the normal limits of a watercourse” [1], flood events have the potential to be highly destructive to communities, both socioeconomically and environmentally [2]. Floods impact the largest number of people across all natural hazards globally, accounting for one-third of all natural disaster casualties and damages [3,4]. The annual expected costs of global floods have been estimated at just over $160 billion (2022 AUD inflation adjusted) [5]. These costs are widely predicted to rise due to the combined impacts of anthropogenic climate change and increasing urban development, especially on floodplains [6].

Australia has a highly variable climate, which results in the country being frequently impacted by flood events [7]. The inter-annual variability in flood occurrence in Australia is modulated by the status of influential climate drivers, but chiefly the El Niño–Southern Oscillation (ENSO) [8]. The negative phase of the ENSO, La Niña, is strongly associated with high occurrence of flood events, as it typically results in wetter conditions over large parts of Australia [9]. Significant Australian floods of the last decade, including the 2011 Queensland flood, as well as the 2021 and 2022 eastern Australian flood events, have all occurred during La Niña events.

Consequently, flood risk and damages have been found to increase in Australia during La Niña periods [10]. This has manifested in infrastructural losses of over $2.4 billion (2022 AUD inflation adjusted) in the 2011 Queensland flood [9]. More recently, widespread flooding in early 2022 took 22 lives and caused devastation to communities across New South Wales and Queensland, the exact financial cost of which is yet to be determined.

Climate projections suggest Australian flood events will likely be more frequent as impact of anthropogenic climate change increases. The Intergovernmental Panel on Climate Change (IPCC) reported that the frequency of heavy rainfall and flood events is likely to increase in the Australasian region (medium confidence) due to climate change [11]. This comes as a result of increased atmospheric moisture fluxes due to higher rates of evaporation and evapotranspiration.

An additional contributing factor is increasing urban floodplain development, which is known to be linked to worsening flood damage [12]. Wider research notes the correlation between this combined anthropogenic influence and elevated flood risk globally [13,14]. Additionally, ref. [15] found that the slowing of Atlantic Overturning Circulation would result in permanent La Niña-like conditions if collapse were to occur, meaning Australia may have permanently heightened flood risk. This underscores the need for a strengthened system of evidence-based flood risk assessments (FRAs) that consider all perspectives in order to proactively mitigate the effects of rising flood risk.

There are three major types of flooding: riverine (fluvial), flash flooding (pluvial), and coastal inundation flooding. This study focuses on inland flooding (fluvial and pluvial) and does not consider coastal inundation, because coastal flooding is caused by storm surge, which is a separate hazard to flooding resulting from rainfall. This research uses indicator-based methods to investigate fluvial and pluvial hazard risk.

### 1.2. Risk Assessment

The risk of a natural hazard occurring is defined by the IPCC as: “the potential for adverse consequences for human or ecological systems, recognising the diversity of values and objectives associated with such systems” [16]. In modern literature, natural hazard risk is typically determined as the product of risk components: hazard, exposure, and vulnerability. This holistic characterisation of risk captures the complex interplay between the hazard an area is subjected to and the innumerate characteristics of that area that affect how that hazard is experienced. This concept is one that was first introduced by the United Nations Disaster Relief Organisation in 1979 [17] and further developed by Crichton [18]. Nowadays, it is common practice to apply such an approach, with many well-known risk indices, such as the Disaster Risk Index, founded upon this approach (e.g., [19]).

Proactive risk assessments are an important component of risk management, the ultimate goal of which is to reduce disaster risk and losses. They are a crucial step in the development of adequate mitigation strategies (e.g., [20]) because such assessments highlight the most exposed and vulnerable areas to the given hazard. This allows relevant decision-makers and stakeholders to make informed decisions on resource allocation on both short- and long-term time scales.

FRAs are objective and quantitative evaluations of the risk a region faces to the impacts of flooding [21]. These assessments focus on the level of potential loss and the probability of these losses occurring based on the interplay between flood hazard, exposure, and vulnerability [22]. The specific output this creates varies across the literature, but will often involve some combination of hydrological modelling, indicator-based methods, geographic information systems (GIS) software, and remote sensing (RS) to create models, indices, and maps, respectively [23,24]. These outputs ultimately provide flood risk data to users to enable them to identify and address areas of high risk.

### 1.3. Defining Flood Hazard

As with risk calculation, there is considerable variability and inconsistency amongst definitions for risk and its components. This variability is noted by authors of other relevant literature, such as [25], which underscores the need for formal and clear definitions of the important terms. Thus, this research adopts relevant IPCC definitions for risk and its components. The IPCC’s comprehensive definitions have been selected due to their reputable prominence in this subject space. Adopting these definitions for flood risk, hazard, exposure, and vulnerability ensures consistency. In this study, the definition for hazard has been adopted and subsequently adapted for a flooding context. Therefore, this study defines flood hazard as: “the potential occurrence of a natural or human-induced physical flood event or trend that may cause loss of life, injury, or other health impacts, as well as damage and loss to property, infrastructure, livelihoods, service provision, ecosystems and environmental resources” [17].

### 1.4. Risk-Assessment Methods

FRAs can be broadly divided into two major categories: those based on numerical flood modelling or simulations, and those based on some form of indices and risk quantification [26]. However, this distinction is nuanced due to the overlap that can occur between these categories, as some researchers utilise modelling methods to supply data for indices (e.g., [27]). GIS software and RS are separate tools frequently used in tandem to support FRAs [28]. These tools are widely considered to combine together to “facilitate an excellent understanding of a region’s hydro-morphologic characteristics” [29], and thus are essential aspects of FRAs.

Hydrological models can accurately simulate hydrological processes and flood inundation with a range of models usually working on small spatial scales [30]. This method of FRA often produces data and maps that describe flood behaviour characteristics, such as flood depth or extent, flood velocity, flood duration, and finally flood hazard. In this type of FRA, flood hazard is typically calculated as the product of flood depth and velocity. Because the hydrological models compute on such a small spatial scale (as flood behaviour will vary strongly over small distances), they are typically resource-intensive and therefore difficult to use at larger scales [31]. A key drawback of this approach is that these assessments are typically flood ‘hazard-centric’ assessments, which pay less attention to the exposure and vulnerability aspects of risk. In this sense, modelling-based methods can be considered a less holistic approach to FRA than indicator-based methods.

Indicator-based approaches use multi-criteria decision-making (MCDM) techniques to geospatially quantify the risk in a region [32]. This can involve standardising geospatial data into several indicators based on the risk components (becoming a number ranging from 0 to 1), and then combining them into a final risk index [33]. In instances where flood modelling data are not used, these assessments are considered a proxy form of FRA. In order to decide the weighting of the numerous indices and subsequently equate them, a form of MCDM is used, the most common forms being fuzzy logic and the analytic hierarchy process, as well as other machine-learning models [32,34,35]. Fuzzy logic, a highly popular MCDM method, computes the degree of relative truth and falsehood as a number between 0 and 1, commonly equating to a probability [36]. In a risk-assessment context, this degree of truth corresponds to the level of risk. By numerically quantifying the risk components, indicator-based proxy approaches using MCDM techniques can holistically assess flood risk [34,35]. This is the method used to quantify flood hazard in this study.

### 1.5. Australian Risk Assessment

Indices and MCDM methods are not widely used to assess Australian inland (non-coastal) flood risk and, to the best of our knowledge, include only two relevant studies: those being [35,37]. These earlier studies are both founded upon the traditional natural hazard risk components this research is utilising (hazard, exposure, vulnerability), and they both use fuzzy MCDM methods for data standardisation and overlay, albeit in different ways. However, the research by [37] differed from this research due to its focus on urban areas, which subsequently featured a comparatively very small study area approximately 1/1000th the size of the study area in this research. The research done by [35] was conducted over a similarly small study area and used a rare set of 24 indicators and a relatively novel ‘fuzzy inference system’ of standardisation. Despite indicators being a common tool for natural hazard risk assessment, there is limited application in an Australian flooding context [33].

However, the Australian FRA space is dominated by modelling-based methods. One such example of this type of study is by [38], assessing the Scenic Rim Local Government Area (LGA) in Queensland, Australia. These FRAs are predominant because they are conducted at an LGA or river-catchment level in accordance with [39], an Australian Government handbook. As already mentioned, this type of risk assessment tends to be highly resource-intensive and has the potential to overlook critically important exposure and vulnerability elements of risk. This resource-intensive nature means that these studies are limited to small spatial scales. As larger study areas are widely unseen in Australian FRAs, this is considered a research gap. Thus, an FRA predominance of modelling-based assessments leaves little room for alternative perspectives, which can result in the loss of important information that other less popular assessment types may produce.

Australian FRAs have little representation in the literature. The prevailing approach to Australian FRA created by the AIDR guidelines has meant that indicator-based MCDM methods are seldom used to assess flood risk in this country. This broader research is founded upon the notion that local decision-makers can benefit from the additional information leveraged by indicator-based FRAs because they are more balanced assessments of an area’s flood hazard, exposure, and vulnerability, potentially from this aforementioned alternative perspective.

In this case study, we used an indicator-based method to assess and map inland flood hazard risk in Australia’s Hawkesbury-Nepean catchment (HNC). Considering this context of Australian FRAs, this research has two major aspects of novelty. Firstly, this FRA was conducted over a large, catchment-wide scale that is greater than the LGA or smaller scales that are typical of Australian FRAs. Secondly, this assessment was conducted using proxy indicator methods, the usage of which are highly limited in Australia. Using this method means that the developed index could be easily replicable and scalable and not be spatially constrained by typical resource-intensiveness issues.

The paper is organised as follows. Section 2 presents descriptions of the study area, indicators, and their respective datasets, as well as the method of creating this index. Section 3 contains a detailed description of the results, including maps of each indicator and the flood hazard index. These results are then discussed in Section 4, and finally the key achievements are detailed in the conclusion.

## 2. Materials and Methods

### 2.1. Study Area

**Figure 1:**
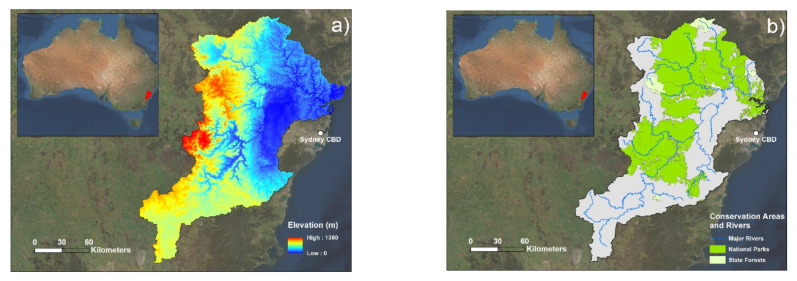
Maps of the Hawkesbury-Nepean catchment study area: (**a**) elevation data (m) of the catchment, and (**b**) the location of conservation areas as well as major waterways.

The study area was the HNC located west of Sydney, New South Wales, Australia. This catchment is a large area covering over 21,700 km^2^, spanning from Greater Western Sydney to the Blue Mountains and stretching vastly in the north–south direction, from beyond Goulburn to Newcastle (Figure 1; Appendix A presents a map with LGA divisions). The selected study area is regarded as one of the highest flood risk areas in New South Wales, if not Australia [40]. Flood events in this area are highly impactful, the average annual cost of which has been estimated at $70 million [41]. Flood events are frequent in the HNC, as its river systems are regularly inundated with more water than can exit the system in a timely manner, with dramatic backwater effects over a large floodplain area. This has been attributed to a combination of large upstream catchments (such as the Warragamba Dam) and narrow sandstone gorges downstream [40]. Figure 1 highlights the physical elements of this area that make for general high flood risk: areas of high elevation draining into a low and flat floodplain, as well as a high number of tributary rivers that drain into these major rivers in the floodplain area. Recent flood events in both 2021 and 2022 highlight the propensity for regular flooding in the HNC.

Specifically, the scope of this research entails using data from the March 2021 flood event as a temporal case study: time-dependent data (rainfall and soil moisture) comes from within this period. This flood event occurred from 17 to 26 March 2021. Large parts of eastern Australia experienced devastating floods that killed five people and forced 18,000 to evacuate. Heavy rainfall and wet antecedent conditions influenced by the 2020–2021 La Niña event were largely attributed to causing this flooding to occur [42]. Persistent heavy rainfall resulted in the Warragamba Dam, the major dam in the HNC, to release over 450 gigalitres of water per day for several days. This resulted in an estimated $65–97 million in insurance claims alone in this catchment [41]. It is logical to anticipate an increase in flood hazard risk across the catchment during this 9-day period, given that this was when extreme rainfall and high soil moisture was experienced. These are two of the three inputs that are explored in this research.

### 2.2. Methodology Overview

**Figure 2:**
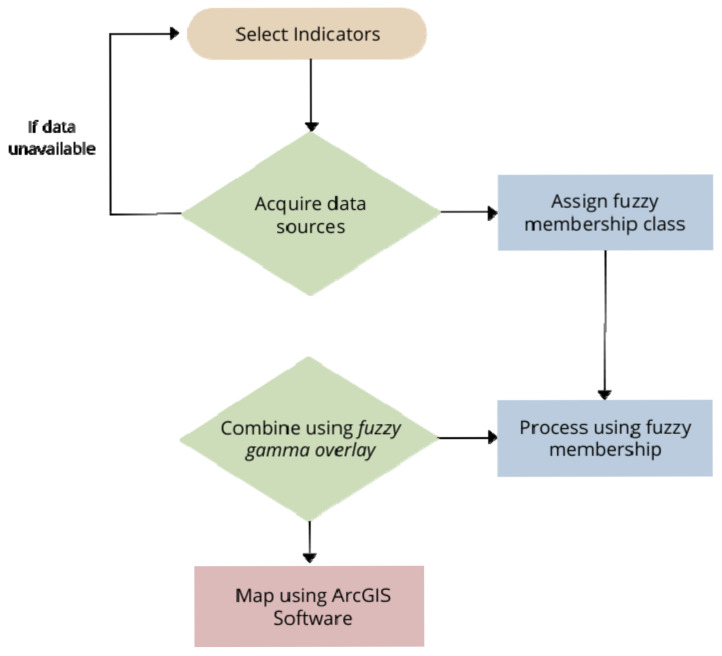
Flowchart of the methodology of this research.

The methodological steps proposed for this research are outlined in Figure 2. These are the steps that should be followed in order to replicate this research.

The novelty of this method lies within the indicators selected and the respective datasets utilised, as the proxy nature of the indicators that should be selected are the key to ensuring that this FRA methodology can be completed over large spatial scales.

### 2.3. Indicator Selection and Creation

The selection process for the indicators to be used to create the flood hazard index (FHI) was a combination of two major factors. Firstly, information from prior literature with similar methodologies (indicator-based MCDM method studies) was used to gain an understanding of the typical indicators used. Secondly, localised input based on the characteristics of the HNC study area was incorporated. For example, soil moisture has been selected as an indicator as it is a representative of the antecedent conditions of this region, something that was identified as critical to the occurrence of flooding in Australia [43]. The following section will explain the justification for each indicator’s selection.

Earlier studies, such as [35,44], used a large number of indicators for their indices. Inspiration was drawn from this when selecting indicators for this research. Whilst drawing inspiration from such studies as these, this research used only three indicators to quantify flood hazard risk. This was for two main reasons: firstly, when a greater number of indicators are used to create an index, there tends to be increasing overlap among these indicators, which ultimately results in unnecessary data processing. For example, [44] utilise both ’river distance’ and ‘waterway and river distance’ indicators, which have only minor difference and thus can be considered to have unnecessary overlap. Other hazard-related indicators seen in similar flood risk index studies included ‘topographic wetness index’, ‘normalised difference water index’, ‘stream power index’, ‘flooded area ratio’, and ‘annual rainfall’ [29,32,35,44]. These indicators and others were considered, but ultimately not utilised in this research because—in comparison to those described in Section 2.3.1, Section 2.3.2 and Section 2.3.3—they are either less widely used, less directly relevant to the study, or they would overlap with other similar indicators that could be chosen.

#### 2.3.1. Maximum 3-Day Precipitation

The maximum 3-day precipitation (M3DP) indicator describes the total accumulation of rainfall in a given 3-day period. This indicator can either be created using rain-gauge data or satellite-estimated precipitation data. This research used the latter, due to the desirable gridded and globally popular nature of this dataset. Earlier studies usually included rainfall as an indicator (particularly annual rainfall) [29,32,35]. It is also common to see 1 day used as the time interval (e.g., [44]). However, given the conscious dual focus on pluvial flooding and longer-onset riverine flooding in this research, a 3-day period was chosen as the interval to cover both anomalous single-day and multi-day rainfall events. This was also to capture a greater proportion of the event’s extreme rainfall, as 1 or 2 days may have been insufficient in this regard. Given that a 3-day period was not observed in the relevant literature for precipitation-related indicators in Australian FRAs, this aspect of the index is considered an element of novelty. This indicator was quantified using rainfall data in the HNC for consecutive 3-day periods during the March 2021 flood event.

Satellite precipitation data were provided by the World Meteorological Organization’s Space-Based Weather and Climate Extremes Monitoring (SWCEM) [45]. The GSMaP (Global Satellite Mapping of Precipitation) space-based precipitation product was used in this study, as it demonstrated high performance over Australia [46,47]. The M3DP dataset was created using a Python programming script. Satellite precipitation daily data were combined into groups of 3 consecutive days in the form of a downloadable NetCDF file. These groups of 3 days covered the entire flood event to capture all rainfall, 3-day totals ending on each individual day from 19 March 2021 to 26 March 2021. This produced a series of 3-day precipitation accumulation maps that sequentially covered the duration of the flood event and illustrated the progression of rainfall. From this, a combined map was made of the greatest 3-day accumulated rainfall at each grid point during the flood event, regardless of the specific 3-day period it came from. This was done so that the indicator quantified the highest 3-day precipitation accumulation at each specific grid point during the flood event, as this ultimately relates more strongly to flood hazard than using any consistent 3-day period for every point in the study area. This combined gridded data set of the highest 3-day precipitation accumulation during the flood event was the final input used for the M3DP indicator.

#### 2.3.2. Distance to River—Elevation Weighted

The distance to river—elevation weighted (DREW) indicator quantifies the distance of each point over the study area to the nearest major waterway. Given the HNC has several major rivers and tributaries that are prone to bursting their banks, the flood hazard of each point in this region will vary considerably based on its proximity to a nearby waterway. When these waterways are overcome with rainfall, the points closest to the river will be the earliest to experience the flood hazard. The more generic DTR indicator is frequently used in this type of FRA: it is referred to as “one of the most significant parameters that can be applied in flood risk mapping” [44]. In this study, the DREW indicator incorporates its own element of novelty, because the DTR layer was combined with an elevation layer prior to standardising to add robustness to the risk values given to each part of the river. This is because the lower-elevation areas will tend to have more water flow and thus be more hazardous, and hence weighted as more hazardous when the same distance to the watercourse.

The DREW indicator was created using ArcMAP10.7 software. This involved downloading a shapefile of the HNC area and the NSW rivers from ArcGIS online via the BoM Geofabric (Table 1). The ‘Euclidean Distance’ function in ArcMAP10.7 was used to determine the distance of each pixel to the nearest river within the HNC area. However, it was noted that using the simple ‘Rivers’ layer alone was equally weighting all parts of a river. This was considered to be misrepresentative of this river system because the downstream and lower-elevation sections of the rivers are much more hazardous and flood prone than the upstream sections, which are typically smaller and flowing less. To address this issue, the first ‘Distance to River’ layer was then standardised and multiplied with an elevation map in ArcMAP10.7. This then created a map of the distance of each point to the nearest river weighted by elevation.

#### 2.3.3. Soil Moisture

The soil moisture (SM) indicator is a gridded data layer that quantifies the moisture level of the soil across the HNC. As mentioned in Section 2.2, SM has been selected as an indicator as it represents the antecedent conditions of the region prior to the flood event. These antecedent conditions are crucial for the HNC, as they largely modulate the impacts of heavy-rainfall events and can hence make them more hazardous. For example, in February 2020, parts of the catchment received over 450 mm in the week ending 13 February. This is very similar (albeit slightly less intense) to the rainfall received in March 2021 that caused widespread flooding in the catchment. However, the dry antecedent conditions of 2020 resulted in heavy rains being received on drier soils and lower dam levels, and flooding did not occur. The effects of a lasting La Niña event 1 year later meant the landscape was much wetter (and in some cases near saturated). The dam and SM levels were higher, enough to flood when a similar rainfall event was received.

Although not a common indicator in this form of assessment, SM is evidently one key antecedent factor, the quantification of which will serve as a representative for wider conditions. Research by [43] highlights the importance of antecedent catchment conditions in an Australian context, further justifying the use of such an indicator. This is yet again an element of innovation to this form of proxy FRA in an Australian context, meaning that all three indicators are founded with their respective element of novelty. In this study, SM was quantified as the 30-day average prior to the event.

The SM dataset was created using the BoM’s AWRA-L (Australian Water Resources Assessment Landscape) daily gridded absolute root zone soil moisture data model [48]. These data measure the amount of volumetric water per unit soil in the top 0–100 cm of the soil profile and are available to download from the Australia Water Outlook, the BoM operational soil moisture modelling dataset. In order to calculate the 30-day average prior to the beginning of the event, a Python script was used to combine 30 individual days of data into an average gridded value across the study area. This gridded average value, calculated from 18 February 2021 to 17 March 2021, was the final input used for the SM indicator.

### 2.4. Data Collection

Data collection and indicator metadata are presented in Table 1. All data use is licensed under the Creative Commons CC BY-NC 4.0 licence.

### 2.5. Data Standardising

The data for each of the indicators exist in different formats, sizes, and units, and are measured with different scales (e.g., rainfall data up to 255 mm (M3DP), river distances up to 31,000 m (DREW), and absolute SM% between 0 and 100). As the various data were in various spatial resolutions (see Table 1), all data were resampled to 50 m grid-size resolution prior to standardisation. A standardisation process was required in order to accurately compare the data and ultimately combine the three indices into a final FHI. The data were standardised using fuzzy methods via ArcMAP10.7 software, a commonly used standardisation method in natural hazard (including flood) risk assessments using MCDM methods (e.g., [32,35,49]). To do this, indicators were assigned a fuzzy membership class. This describes the relationship between the indicator and flood hazard risk. In this case, SM and M3DP have a positive relationship with flood hazard risk, as greater values of SM and M3DP were more likely to contribute to greater flood hazard risk. Therefore, both were assigned to the fuzzy large class. The inverse is true for the DREW layer, whereby there exists a negative relationship between this indicator and flood hazard risk. This means the lower the value of the DREW indicator (the smaller the distance to river), the higher the flood hazard risk. Therefore, the fuzzy small class was applied in this case.

To accurately represent the hazard risk in context of temporal precipitation and SM conditions, historical averages of the M3DP and SM parameters were used as the midpoints for the fuzzification process. The average M3DP for a given March day was calculated using the same GSMaP satellite data to ensure consistency, using daily averages for the month of March since records began in 2000. This produced a 90th percentile value of 27.5 mm in a given 3-day period. The 90th percentile value was used for the fuzzy midpoint as a representative of a statistically high value to compare to as a baseline for comparison. Similarly, the average March SM value was calculated using historically modelled AWRA-L SM grids dating back to 1900 for its respective fuzzy midpoint, which was 36.65%. This average was also calculated using the same dataset as the rest of the indicator (see Table 1). The result of this fuzzification process was that each of these indicators was standardised to a dataset with values ranging from 0 to 1, whereby factors that are more likely to contribute to flood hazard risk (larger values in fuzzy large sets and smaller values in fuzzy small sets) were weighted more strongly.

### 2.6. Index Calculation

In order to calculate the final FHI, the Fuzzy Gamma Overlay function within ArcMAP10.7 was used to combine the three indicators. Fuzzy Gamma Overlay multiplies indicators together whilst ensuring the result is standardised between 0 and 1. Fuzzy sum and fuzzy product methods are multiplied and taken to the power of a gamma value. Equation (1) describes this process, whereby γ is the gamma value and µ is the indicator. For this research, the generic gamma value of 0.9 was used—this being the industry standard. The use of this method means that all indicators were equally weighted, which is an objective index-creation approach without further modelling.

µ(gamma) = (FuzzySum)^γ^ × (FuzzyProduct)^1 − γ^, (1)

### 2.7. Correlation Analysis

Correlation analysis between each of the three indicators and the final FHI was undertaken using a Pearson correlation function in Python. This function generated correlation values between each raster layer and the final index.

## 3. Results

Both the M3DP and SM indicators are presented with a series of three maps (Section 3.1.1 and Section 3.1.3). The first maps are a visualisation of the raw data (in mm for M3DP and a percentage for SM). The second maps are calculated using only data from the event to illustrate the spatial variability within the March 2021 case study, and the third maps use historical context in standardising to demonstrate the magnitude of this flood event compared to the past events. This historical context is a comparison to a 20-year average for M3DP and a 120-year average for SM. Similarly, the DREW indicator is presented with two maps: a pre- and post-fuzzification illustration.

Standardised indicators and the final FHI were mapped using a standard set of quintile values. These quintile values were then assigned a class of risk (very low, low, moderate, severe, extreme) (Table 2). The classification breaks on the maps were then manually adjusted to these classes.

### 3.1. Indicator Maps

#### 3.1.1. Maximum 3-Day Precipitation

**Figure 3:**
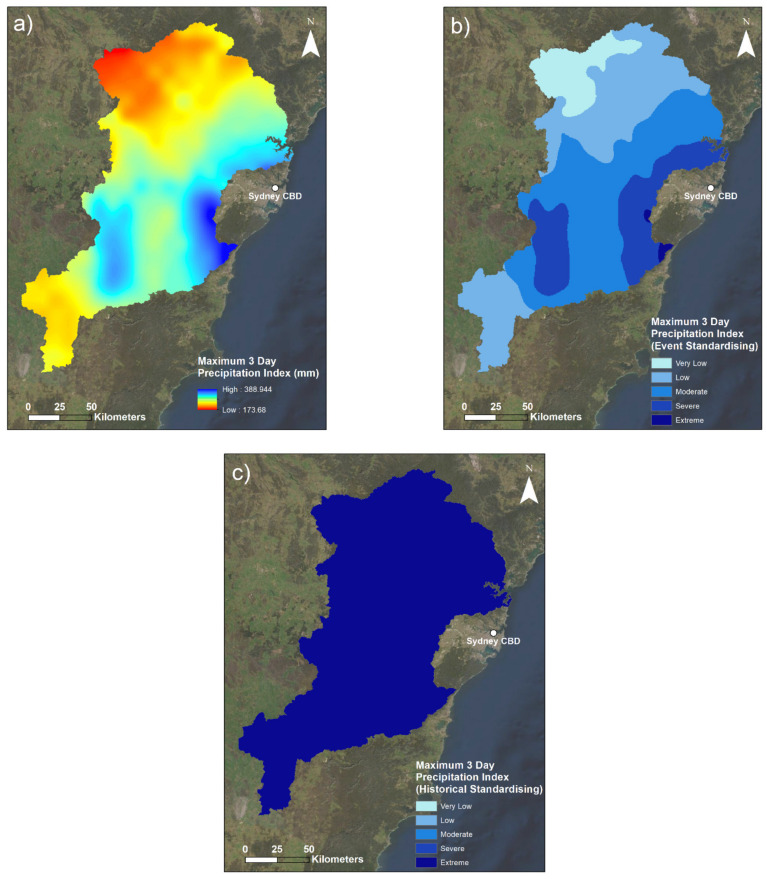
Maps of the maximum 3-day precipitation indicator: (**a**) the raw maximum 3-day precipitation accumulation values; (**b**) the indicator with spatial standardising solely across the flood event; (**c**) the indicator after being standardised spatially and temporally with respect to historical March 3-day rainfall events. Darker areas in (**b**,**c**) indicate greater maximum 3-day precipitation risk.

Maps of the maximum 3-day precipitation indicator are presented in Figure 3.

Figure 3a,b illustrate the spatial variability of the maximum rainfall received in any given 3-day period during the flood event. One can note the clear trends of increased rainfall towards the coastline in the Sydney direction and particularly around the central south-eastern part of the catchment area. This pattern is supported by the synoptic conditions during the time of this event: a blocking high pressure system in the southern Tasman Sea resulted in prolonged easterly onshore flow over this area [42]. Onshore flow during the event meant the majority of the rainfall fell closest to the shoreline, before decreasing in the inland direction. This synoptic pattern is reflected in the data. Although Figure 3a depicts the north-west corner of the catchment as the lowest ‘red’ colour, and Figure 3b describes this risk as comparatively low for the event, this area received at least 173.7 mm in a consecutive 3-day period. This clarification is important because 173.7 mm is still a very large amount of rainfall in a 3-day period, and Figure 3c helps provide a similar perspective to this.

The purpose of including Figure 3c is to highlight the extremity of the rainfall of this event compared to past events. This figure depicts that the entire study area received an extreme amount of rainfall in a 3-day period when compared to historical March rainfall (2000–2020). Whilst the lower boundary of the extreme category is 0.8, it is important to note that all historically standardised indicator data were above 0.94. This further demonstrates the extremity of this rainfall event, and that regardless of the variability in this rainfall displayed by Figure 3a,b, it was extreme throughout the entirety of the study area.

#### 3.1.2. Distance to River—Elevation Weighted (DREW)

**Figure 4:**
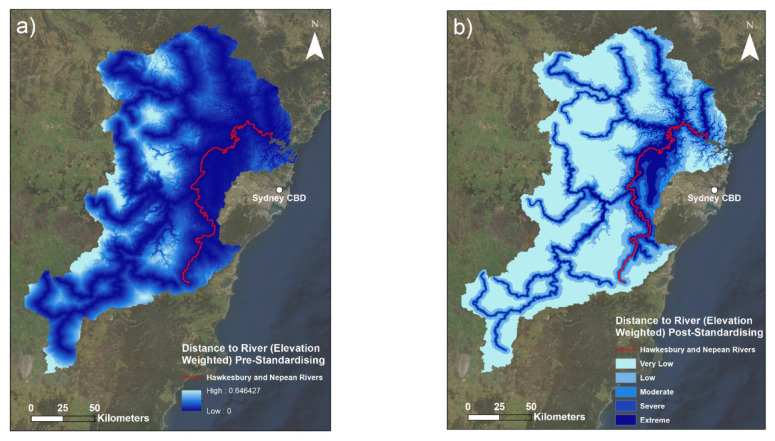
Maps of the distance to river—elevation weighted indicator: (**a**) the raw distance to river—elevation weighted data; (**b**) the fuzzified distance to river—elevation weighted indicator. Lower raw values (darker areas) in (**a**) indicate closer proximity to a river with respect to elevation. Higher standardised values (darker areas) in (**b**) indicate greater risk from this indicator (closer proximity). The major Hawkesbury and Nepean rivers are outlined to illustrate their location.

Maps of the distance to river—elevation weighted indicator are presented in Figure 4.

Creating the DREW layer using a combination of the distance to river layer and an elevation layer had implications for the final product visualised in Figure 4. The elevation layer contains clear outlines of the lowest valleys. This corresponds to the rivers and creeks of this area. Therefore, these smaller water bodies of the area were represented in addition to the rivers, thus making the overall indicator more comprehensive and robust in comparison to regular distance-to-river inputs that do not include this elevation addition. This was practically applicable because the main rivers were classified as the highest level of risk, and the secondary creeks and waterways illustrated by the elevation layer were classified as lower to moderate levels of risk, depending on their elevation. Note that the level of elevation of these secondary waterways have strongly modulated the index values of similarly sized waterways. These waterways in the north-west of the catchment (among the lowest elevation areas) have much stronger values than similarly sized ones in more elevated areas, which seem to disappear almost between the DREW pre-standardised (Figure 4a) and the DREW post-standardised (Figure 4b). This mechanism has also resulted in slightly larger ‘buffers’ of risk (higher risk lines surrounding the river paths) existing around the lower-elevation sections of rivers contained in the river layer and smaller index values at the same distance around higher-elevation river sections.

#### 3.1.3. Soil Moisture

**Figure 5:**
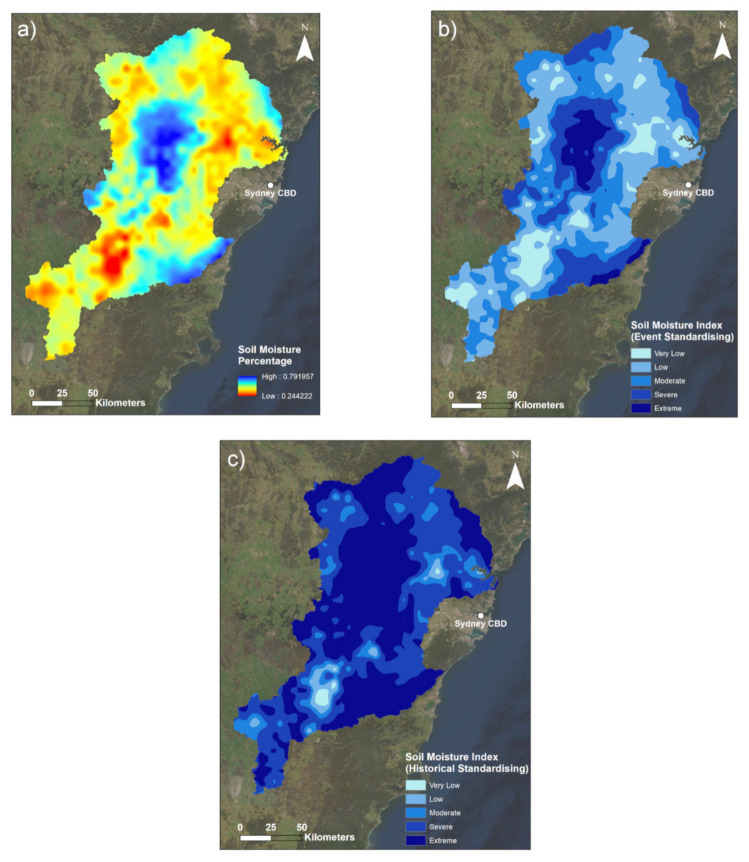
Maps of the soil moisture indicator: (**a**) the raw root zone soil moisture percentage for the 30 days prior to the flood event; (**b**) the indicator with spatial standardising solely across the flood event; (**c**) the indicator when standardised in comparison to the historical March soil moisture. Darker areas indicate greater SM% in (**a**), and greater SM index values in (**b**,**c**).

**Figure 6:**
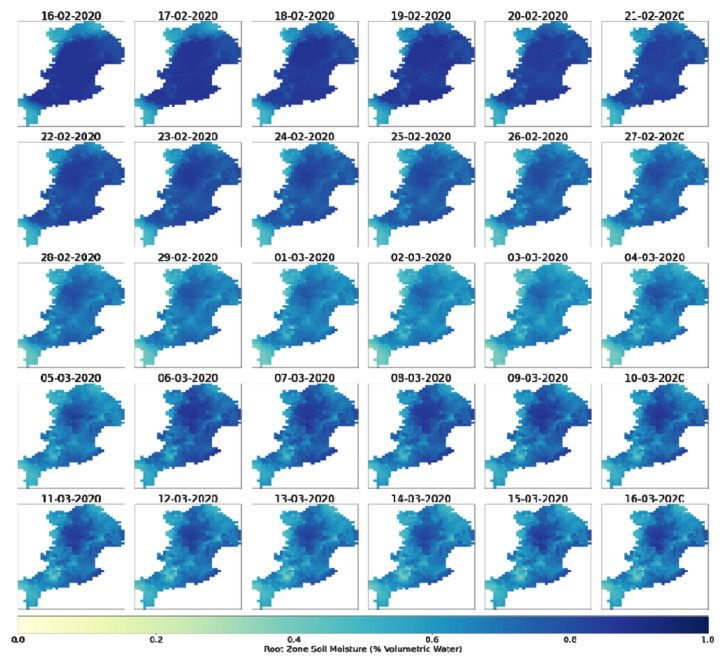
Daily soil moisture time series (16 February 2021–16 March 2021).

Maps of the soil moisture indicator are presented in Figure 5. A daily time series of the Catchment Soil Moisture 30 days prior to the flood event is depicted in Figure 6. 

### 3.2. Flood Hazard Index

**Figure 7:**
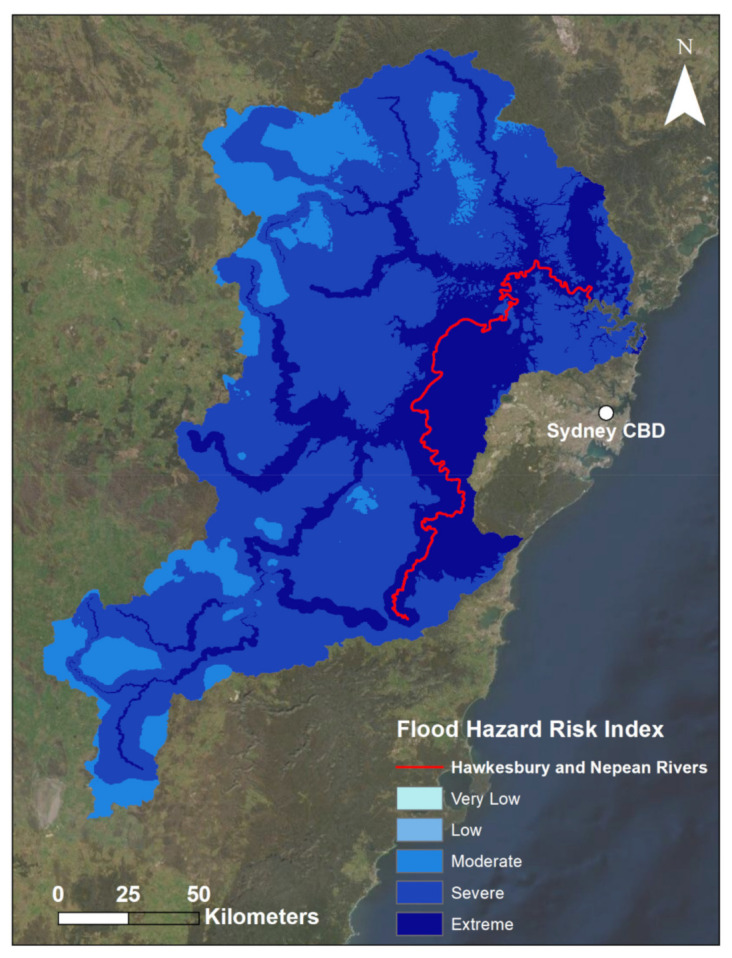
Map of the final flood hazard index. The legend describes the relative flood hazard risk during the March 2021 flood event. Note that SM and M3DP indicator data with historical context were used to create this index. Darker areas indicate greater flood hazard risk. The major Hawkesbury and Nepean Rivers are outlined to illustrate their location.

A map of the soil moisture indicator is presented in Figure 7.

Figure 7 illustrates the clear imprint on the map that the DREW indicator had on this flood hazard index. The observable lines of extreme risk align with the river lines as per the DREW maps in Figure 4. Since there are no areas categorised as very low risk, and only a very small area of low risk, this further underscores the extremity of the conditions of this flood event, particularly with respect to rainfall. Visual inspection does not reveal a great deal of influence from either of the M3DP or SM indicators; however, one can note that these indicators do influence the final index most notably in areas where these indicators are at a minimum. For example, the area of reduced SM in the south of the catchment has visibly reduced the overall risk in that area, which can be seen in the reduced risk of the river layers in that area compared to those surrounding it. Similarly, the area in the north-west of the catchment area with the lowest M3DP values also has reduced risk and a larger proportion of moderate values than the surrounding areas.

Furthermore, given the highly extreme nature of the rainfall during this event, it comes as no surprise that 83.6% of the catchment was classified as either a severe or extreme level of flood hazard risk, i.e., a standardised value greater than 0.60. The extreme areas are primarily located in the eastern floodplain areas of the catchment, which is where the inflow of a large number of tributary rivers to the larger Hawkesbury and Nepean rivers meet. Moderate risk areas are predominantly located to the west of the study area, leaving everything in between to be largely classed as severe risk. Table 3 indicates that low-risk areas occupy only 0.005% of the study area and there are no areas of very low risk. Ultimately, this demonstrates that this risk index over this catchment area during the March 2021 flood event is almost entirely composed of moderate flood hazard risk and above.

### 3.3. Correlation Analysis

Correlation analysis reveals that the DREW indicator was correlated the strongest with the final FHI (0.76, *p* < 0.0001). This correlation value of 0.76 is considerably larger than the other two indicators and shows a strong positive correlation between the DREW layer and the FHI. This supports the visual observations of the FHI map made in Section 3.2. M3DP had the next strongest correlation value of 0.58 (*p* < 0.0001), indicating a moderately strong positive correlation between the M3DP layer and the FHI. Similarly, SM is correlated with a value of 0.16 (*p* < 0.0001), indicating a positive, albeit weaker correlation between the SM layer and the FHI. Potential reasons for these results and implications are discussed in Section 4.5.

## 4. Discussion

### 4.1. Maximum 3-Day Precipitation

**Figure A2:**
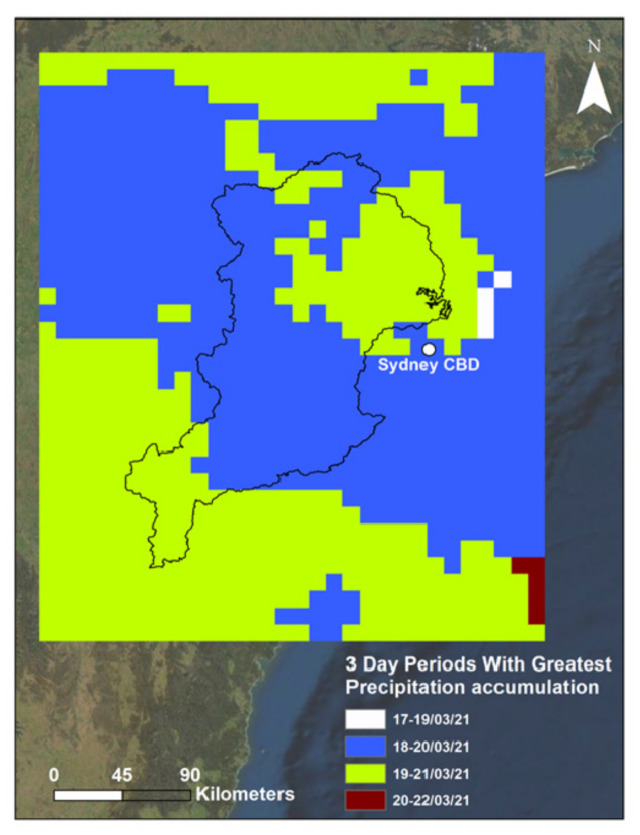
Three-day periods with greatest precipitation accumulation map. This is the direct output of the M3DP indicator, illustrating which grid points used which 3-day period as their maximum.

The M3DP indicator was calculated as the maximum rainfall accumulation in any 3-day period during the flood event (17–26 March 2021) at each 0.1 degree grid point in the catchment area. This means the final map layer consisted of 3-day totals that could have come from any consecutive 3 days in that 8-day period if it was the maximum value for that given grid point. Ultimately, the data comprising the final maps were a combination of two 3-day periods: 18–20 March 2021 and 19–21 March 2021. It was these two 3-day periods that presented maximum rainfall accumulation within the bounds of the catchment. The spatial distribution of which date was used for each grid point is illustrated in Figure A2 (Appendix B). These sets of 3 days received the highest rainfall over the catchment area, recording a maximum of 417.6 mm and 339.6 mm, respectively. Whilst the maximum rainfall fell in those two aforementioned periods towards the start of the flood event, it is worth noting that considerable rainfall totals were observed throughout this flood period: some locations in the catchment recorded over 500 mm for the week ending 23 March 2021. This consistent rainfall caused a second peak in the floodwater heights to occur at multiple locations.

Despite the fact that the entirety of the catchment received extreme rainfall in comparison to historical standards, Figure 3 demonstrates that the eastern and coastal areas of the HNC were most at risk of pluvial flooding (and ensuing fluvial flooding) from solely a precipitation perspective. This area includes the built-up areas of Greater Western Sydney at the central-eastern boundary of the study area, which is particularly problematic for its population-dense communities and corresponding infrastructure in these areas. Furthermore, because the rivers in this catchment largely flow from south to north, the stronger rainfall in the southern part of the catchment shown in Figure 3a tends to have filled tributaries and smaller rivers. This creates the potential for longer-onset river flooding when that water reaches the highest-flowing parts of the major rivers. The observed double peak in flood waters and overall long duration of the 2021 flood event is demonstrative of this mechanism.

Figure 3b was created using the midpoint recorded from the flood event data alone during the fuzzification process, which was 281.31 mm. It is clear that using an event-specific midpoint alone is not reflective of historical rainfall conditions, in that it creates a skew towards smaller values that ultimately makes it difficult for decision-makers to understand this event with respect to others. In comparison, Figure 3c used the historical 3-day 90th percentile midpoint of 27.5 mm, as per Section 2.5. This meant that every fuzzified value in the catchment was contained in the largest standardised class (extreme) and above the value of 0.94. It was found that on average, any 3-day March period would expect to see around 9.4 mm, a 90th percentile period 27.5 mm, and a 99th percentile value of 143.6 mm. Therefore, this means the whole catchment was above the 99th percentile for M3DP (as the minimum was 173.7 mm), and the areas that received up to a maximum value of 417.6 mm were much rarer. From a records perspective, New South Wales had its second-wettest day, third-wettest week and second-wettest March since records began in 1900 [42]. This ultimately highlights that the historical standardising is clearly congruent with the contextual setting and is able to capture the event’s extremity. Thus, the rainfall experienced during this flood event should be considered extreme, if not unprecedented.

### 4.2. Distance to River—Elevation Weighted

Given that flowing waterways are the starting point for any fluvial flood (by definition), the characteristics of a waterway are highly important to understanding its flood hazard. Elevation has long been considered a key characteristic and modulator of stream flow rates, with research dating decades back noting this importance [50]. Therefore, this was the environmental element used in combination with the distance-to-river layer. Large parts of the study area are classified as the lowest category of DREW risk (<0.2). This was to be expected, especially in the more elevated regions to the west of the study area. This is because well-elevated areas that are not particularly close to rivers should not expect to be impacted by fluvial flooding, as the floodwaters would likely be limited by topography, if nothing else. If these areas were to experience any form of flooding, it would be due to flash flooding after an extremely intense rainfall in a short period of time—something that is instead captured by the M3DP within this FRA. The complexity of flash flooding is a common challenge for FRAs, with complicated machine-learning algorithms typically required to model this phenomenon on small scales [51,52]. Conversely, this research uses the M3DP indicator to quantify both flash- and river-flooding risk by proxy.

Figure 4 illustrates that the broadest areas of highest DREW risk are located in the downhill and downstream areas on the eastern side of the catchment, which are areas where water is naturally more likely to accumulate and flood. This is because water will typically flow in greater quantities and at higher speeds in lower-elevation portions of rivers, when other waterways and tributaries have inevitably congregated during the descent. In the case of the HNC, this is particularly relevant due to the large system of tributary waterways illustrated by Figure 2.

The added complexity of the use of the elevation layer also meant that locations which are equidistant from the river but at different elevations were found to have different levels of risk. This is why this addition was used. The weighting effects of the elevation layer are also visualised in the differing size of the buffers around each river, whereby the lower the elevation, the larger the buffer of higher risk values around the river. In terms of flood hazard risk, this is theoretically accurate because the low-elevation sections of the largest rivers will tend to flow the strongest and will thus be the highest risk in terms of river flooding. These river areas will also tend to be the widest parts of the river due to this high flow rate, an additional reason for having larger buffers. This trend may not always be visible due to the broad categories used to classify this map, as any value between 0.8 and 1 will be the same colour, for example. In this sense, some of the complexity in this map may not be displayed due to the use of standard choropleth (colour gradient) mapping.

Figure 4 also highlights that all river areas as well as the most significantly low-lying areas were identified as having the highest DREW index values, largely manifesting in the suburban areas in Greater Western Sydney. Upon inspection, one can observe that the largest consolidated area of highest risk from this indicator coincides with the location of the major rivers in these suburban areas. This observation correlates with the broader emphasis placed on the flood-risk management of the Hawkesbury and Nepean rivers by local authorities, as these are the rivers that run through this eastern floodplain part of the HNC. However, the area also contains South Creek, a significant tributary of the Hawkesbury and Nepean rivers, which strongly contributes to these large values in addition to the lower elevation. This congregation of rivers combined with the overall low elevation of the wider floodplain has created a region with observably high DREW index values.

Additionally, one can note the differences in the size of the buffers of values between the pre- and post-standardising DREW (Figure 4a,b respectively), whereby the river lines in Figure 4a appear to have higher values reaching further in the perpendicular direction from the lines of the rivers than in Figure 4b. The reason for this difference is due to the settings used in the fuzzification process. Table A1 (Appendix C) shows that a lowered midpoint value was used in the fuzzy membership calculation to create Figure 4b. This was done with the intention of reducing the size of these buffers, as the default spread value produced buffers of index values that reached too far from the rivers, further than would ever be actually at risk in even an extremely severe flood event in any study area. However, it is worth noting that this was in part a subjective estimate. Therefore, this reduced midpoint lowered the amount of distance that was converted to higher risk values. This had the additional effect of reducing the buffers of the smaller waterways introduced by the elevation layer to only very small regions of higher risk, which is understandable given their generally lower flow rates. Therefore, not only did the elevation aspect of this indicator modulate river risk values (as aforementioned), but the fuzzy midpoint value was also manipulated in order to represent river risk as accurately as possible, another modulating factor to this indicator. This added robustness meant the final DREW layer was more comprehensive for a river-flooding context than typical river-distance indicators observed in relevant literature. Overall, the DREW indicator was innovative and is considered to be a valuable input into the FHI.

### 4.3. Soil Moisture

Whilst SM data is measured as a percentage, percentages ranging all the way from 0–100% are not expected. This is because SM percentage refers to the volumetric amount of a unit of soil that is pure water. For example, the catchment-wide event average SM value of 50.5% indicates that on average, when considering the soil profile of the catchment’s root zone (0–100 cm), 50.5% of the soil volume was pure water. Whilst 50.5% does not seem very large in a broader sense (as half is not typically considered an above-average value), in the context of SM percentages this is actually very high and considered to be close to saturation for many soils depending on their soil type. In this sense, it would be impossible to record an SM% of 100, as there would only be water. This context is important for understanding SM data correctly.

A historical analysis of the whole AWRA-L (Australian Water Resources Assessment Landscape) model period (1900–2020) indicates the minimum and maximum values recorded in the catchment area to be 14.4% and 73.7%, respectively, with a long-term median of 36.7% and standard deviation of 10.8%. This average was created using the March average values for each of these years. However, the maximum value in the catchment for the 17 February 2021 to 16 March 2021 monthly period was 79.2%, which was greater than the historical maximum. This illustrates the extremity of the high-SM areas observed during this event. Having such a high maximum value has slightly skewed the colour scheme of Figure 5a. For example, yellow areas tend to have connotations to below-average values in the broader scheme of a multi-coloured plot. However, in this case, general yellow areas are referring to values of 35–45%, which is at or above the historical March average for the catchment area. This overall perspective means that comparing the 50.5% event average to the 36.7% March catchment median is now a more meaningful comparison than the differences between regular percentages. The visual manifestation of this difference in SM averages can be seen in the difference in index values between Figure 5b,c.

Figure 5c illustrates the result of the same fuzzification process, however in this instance using historical context in the standardisation process. This meant in this instance the average SM value for the month of March was calculated and used as the midpoint for fuzzy standardisation (36.7%). This midpoint was lower than that used to create Figure 5b, which has thus resulted in elevated index values for the entirety of the catchment area. Therefore, Figure 5c shows that when considering a historical average, a majority of the study area had SM values classed as extreme. Since soils already containing more moisture are not able to absorb as much water as drier soils: this increased SM value compared to the historical average indicates that this majority of the catchment area had antecedent SM conditions that heightened the risk of flooding.

One of the more striking elements of Figure 5 is the stark contrast between SM values recorded between the central and south-east catchment and the rest of the wider majority of the study area. Figure 5a in particular highlights this contrast between the smaller patches of blue high SM values and the surrounding yellow and similarly coloured areas. Analysis of these areas revealed that these they align with regions of conservation areas in the catchment, in particular the Wollemi and Blue Mountains National Parks in the central catchment area, and the Upper Nepean State Conservation Area in the south-east (Figure 2). Given these conservation areas are typically pristine areas with largely undisturbed soils and low/no population, it is understandable that they would have the greatest SM values. This is because in comparison to other urbanised areas, these highly vegetated conservation areas have the highest soil porosity, which allows for a greater infiltration of moisture to the soil [53].

**Figure A3:**
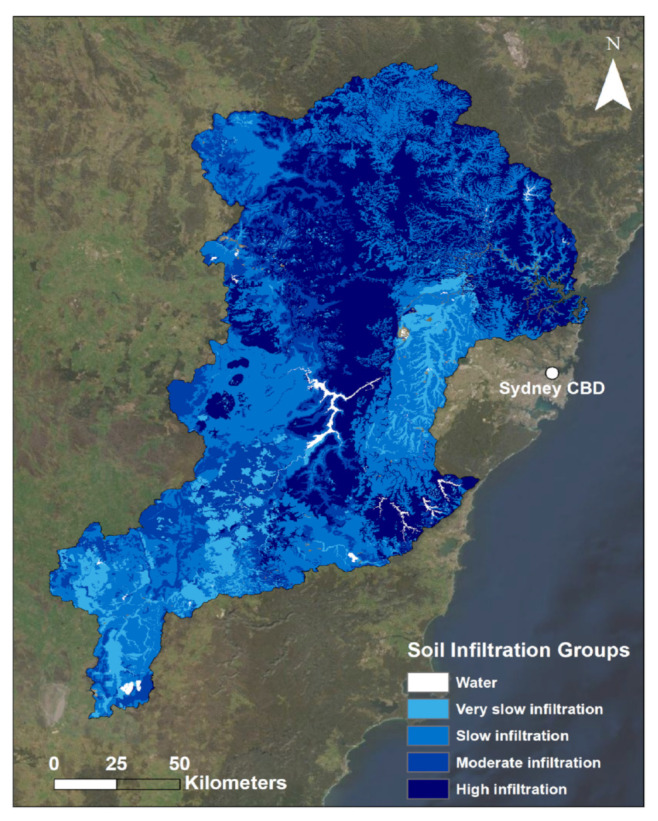
Soil infiltration groups map of the Hawkesbury-Nepean catchment.

Additionally, there is a visually apparent link between the SM of the catchment and the soil infiltration of the respective soil. Figure A3 (Appendix D) presents a map of the soil infiltration levels of the catchment area. These data describes the rate at which water is able to infiltrate the soil, obtained via the NSW Government’s Sharing and Enabling Environmental Data (SEED) initiative. Visual inspection of this layer and Figure 5a,b reveals a link between these areas with high SM values and their soil infiltration rating: large parts of these areas are classified as the highest level of soil infiltration. Similarly, visual inspection of the lowest SM value areas tends to align well with the areas of very slow infiltration. Given how important the rate at which water infiltrates into a given type of soil is to the SM values of that soil, this link is understandable from a physical processes perspective. Analysis revealed a positive correlation of 0.27, indicating that these two elements are indeed linked to a limited extent. Furthermore, the built-up areas of the central eastern floodplain largely show low SM values, and this may be partially influenced by the urbanisation that has taken place in this area. This urban development on the floodplain has undoubtedly impacted the soil’s ability to absorb water through the introduction of large areas of impermeable surfaces and compacted soils [54].

### 4.4. Flood Hazard Index

The creation of the FHI used the fuzzified data with historical context (in the cases of M3DP and SM). Using these inputs means that the overall flood hazard risk of this specific case study event with respect to regular (March) conditions is represented by this data. Furthermore, the use of these fuzzy processes to standardise data and create the final index meant that these index data are sensitive to changes in each contributing indicator. The list of fuzzy inputs to create each indicator can be found in Table A1 (Appendix C), which describes the midpoint and spread values chosen for each indicator to produce the output deemed most accurate. Equally weighting of the three indicators via fuzzy gamma overlay was chosen as it was the most objective method of creating this indicator without further analysis of the inputs. Future research could undertake this analysis further to determine if other weighting techniques should be applied.

Overall, the primary reason for the widespread range of high-risk values observed across the catchment area relates to the extreme nature of this event. As aforementioned, all M3DP historically standardised data were above 0.94 throughout the study area, indicating that the rainfall in all parts of the catchment was above the 99th percentile for 3-day accumulated March rainfall in the catchment. Subsequently, this results in the input from the M3DP data into the FHI ultimately being not extremely impactful on the variability of the final index, as the maximum and minimum values of this indicator are only 6% apart. Similarly, imposing a reduced historical midpoint to the SM dataset meant that a majority of these data was also classed as extreme. These two inputs combined with the DREW layer resulted in much of the variability in the FHI coming from the DREW layer, as this is the only indicator that has a distribution of values with similar amounts of very low and extreme values. This should explain a lot of the trends in this final FHI map It is largely imprinted with the river paths and the visual effects of the elevation input, whilst featuring secondary influences from the M3DP and SM indicators. Considering that this research is focused on river flooding (as well as flash flooding), it is understandable and expected that the DREW layer features so heavily on the FHI. Section 4.5 discusses these respective influences from a statistical standpoint.

In some respects, the broader identification of the floodplain area as having the highest flood hazard risk should be considered a success of this index. This corroborates the aforementioned view of the NSW Government that the Hawkesbury-Nepean Valley has the highest flood risk in NSW, if not Australia [40]. Specifically, this research found that the Campbelltown City Council and Camden Council LGAs had the highest FHI values. Following this were the Liverpool, Penrith, and Blacktown Council LGAs. Crucially, all five of these LGAs were located within this notably high-risk floodplain area. Thus, this research has specifically highlighted these five LGAs as points of concern from a flood hazard perspective, which has the potential to be important for a relevant decision-maker or stakeholder.

Furthermore, widespread parts of this area are indeed those that experienced the most devastating flooding during the 2021 event. This urbanised area to the central-east of the catchment area contains the majority of the human population, but is also where the majority of the flood risk-management resources are allocated. These Hawkesbury and Nepean rivers are the dominant hazardous rivers when it comes to flooding in this catchment and this index has identified them as such. This is not to say that damaging flooding does not occur elsewhere in the catchment, but that the Hawkesbury-Nepean Valley is the key area of interest for authorities and is routinely impacted by flood events with the power to devastate population-dense local communities.

Additionally, since this valley area had comparatively lower SM values, the findings of this research reveal a potentially useful piece of evidence for local decision-makers. That is, any flood-inducing rainfall event originating from onshore flows inwards from the Tasman Sea (as was the case in this event) should be alarming for local authorities, even if antecedent SM conditions are near normal. As aforementioned, the inland-travelling rain bands will cause more rain to fall over the eastern valley part of the catchment and closer towards the coast in the Sydney direction. Therefore, since this valley area recorded some of the highest risk values of this index despite comparatively lower SM values, this research has produced evidence highlighting the risk of this synoptic environment, particularly during phases of La Niña, which typically produce more rainfall in this region. Since these synoptic situations are forecastable, this evidence could be incorporated by relevant authorities into early-warning systems in addition to broader flood risk-management strategies.

### 4.5. Correlation Analysis

Analysis revealed that correlation of all three indicators with the final flood risk index was statistically significant (*p*-value of <0.0001). An M3DP correlation value of 0.58 with the FHI is a moderate value inferring a moderate level of influence over the final index. As one might expect from an input that is all extreme values above 0.94, this will align well in extreme places and not in others. Given that 22.7% of the catchment area has the final hazard risk classification of extreme, all of these regions will be well correlated with the extreme M3DP input. On a macro scale, both the M3DP and hazard layer exhibit the broader trends of higher risk and more Extreme values located to the eastern side of the catchment area, and the lower (more moderate) values lying to the western side of the catchment, besides the smaller region in the south-west. The DREW input also exhibits a similar trend to a certain extent, but this is the trend hypothesized to be correlating to the M3DP layer to its moderate degree in addition to linking well to all extreme areas.

An unsurprising element of this correlation analysis was the strong correlation between DREW and the FHI. A value of 0.76 highlights that this indicator had a relatively strong influence on the hazard risk output. As aforementioned, a potential reason why this indicator has such a strong influence is because it is the only input with a wide distribution of values, meaning it is able to spatially modulate the ensuing data better than the other inputs. It is also the only static layer across all events, whereas the M3DP and SM indicators will vary from event to event. Overall, a major reason why this broader result is expected is due to the typically highly influential nature of this indicator as demonstrated in the literature. Studies such as [44,55] found their distance-to-river indicator to be both within their three strongest contributing inputs (of studies with >10 indicators), alluding to the wider view that distance-to-river layers are typically influential in MCDM studies. These layers will always have strong spatial variability and a complete distribution (as they range from 0 to as far away as possible in the study area) where other inputs may not, which is potentially why this result is a common one.

An additional aspect of interest to this correlation was the logarithmic-appearing distribution illustrated in Appendix E. This indicates that as DREW begins to increase, the hazard index rises quickly initially but then tapers off and has diminishing returns as DREW values reach their maximum. One potential reason for this occurring could have been that a reduced fuzzy membership midpoint was used, meaning that the point at which values are standardised towards zero was lower than average. This midpoint of 0.15 approximately aligns with the point at which the correlation values begin to quickly drop towards zero, which could have potentially created this logarithmic-like effect.

An SM correlation value of 0.16 indicates a weak positive correlation with the hazard risk index. Potential explanation for a wider lack of relationship is due to contrast observed between key areas of SM and the corresponding broader trends illustrated by M3DP and final index. Figure 5a in particular visualises this contrast clearly. Crucially, SM does not correspond well with the broader areas of higher risk, namely these aforementioned areas in the HN Valley. These regions for the most part display relatively lowered SM values, and it is potentially because of this general contrast against the broader trends that this SM input is correlated weakly with the FHI. Beyond the aforementioned elevated values of some conservation areas, the indicator displays complex variability against broader trends potentially due to the dynamic and variable secondary factors related to the soil (beyond precipitation alone) that influence SM. Therefore, this could further contribute to a weaker correlation with the FHI.

It is important to note these correlation values (particularly SM and M3DP) would be subject to potentially significant changes depending on the event. For example, in the case of this event, lower SM values occurred in the highest overall risk area, likely due to a variety of different factors that have the potential to modulate SM content, such as temperature, evapotranspiration, preceding rainfall, and soil porosity in that region. In a separate event, if SM conditions were different, and perhaps even higher for regions with high DREW values, correlation values may have been significantly different. Future research could explore a sensitivity analysis in order to make concrete statements regarding the influence and quality of every input in this research, as well as to how they would change under different circumstances; however, this is beyond the scope of this study.

### 4.6. Comparison with the Literature

An earlier study [44] presents an FRA with 11 indicators spanning flood hazard, exposure, and vulnerability elements. This evaluation of index importance found that waterway and river distance (WRD) and river distance were the two most important inputs to the flood risk index. Given that it was noted earlier in Section 2.3 that these inputs are considered overlapping, this means that their river distance indicator is effectively the most important. This parallels the finding of this study that the DREW input correlated the strongest with the flood hazard index, a somewhat similar metric to importance. After these two inputs, [44] found the next most important to be their maximum 1-day precipitation input, which also mirrors the correlation finding in this study, as M3DP was the second best correlated. This similarity also had a visual manifestation whereby both studies were largely visually influenced by the location of the waterways. This is common in FRAs that use a DTR indicator, e.g., [32,56].

Ref. [32] is another similar FRA in methodology that used a DTR indicator that imprinted on the final output heavily. Their hazard analysis revealed that 37% of their study area was classed as the highest risk level, which is not dissimilar to the 22.7% of extreme classification in this particular study. However, due to the differences in study area size, this translates to 843 km^2^ of very high flood hazard risk in [32] compared to 4941 km^2^ of extreme flood hazard risk in this research. From this, one can note the utility of assessing such a large study area, as this research was able to identify a much greater area at higher risk during the study event compared to [32]. This novelty is particularly important is a country as large as Australia, whereby the ability to assess larger study areas will make identifying hazard and broader flood risk across the country a less resource-intensive process.

### 4.7. Limitations and Future Research

This form of FRA often incorporates an indicator that directly represents typical flooding in the study area (e.g., [32,34,57]). Having a flood-related indicator adds to the robustness of a flood hazard assessment. However, some studies choose to use other relevant factors to achieve this standard with a more proxy-related approach (e.g., [42,56,58]). Additionally, given that the HNC is subject to such regular flood events, this study considered to include a flood-related indicator (flood height). Because the HNC is such a large area (Section 2.1), there are no pre-existing flood datasets that cover the entire catchment area. This meant there was ultimately no applicable flood data able to cover this area. However, this absence also meant that a larger area was able to be assessed, which is both a strength and an aspect of novelty of this study, as areas of this size are typically not assessed in Australian FRAs. Furthermore, flood modelling data for this study area may be utilised in future research as a smaller scale case study using this same method, as well as for potential validation of this index. Thus, using the three indicators of distance to river—elevation weighted, maximum 3-day precipitation, and soil moisture suggests satisfactory robustness for a proof-of-concept proxy hazard study of this large spatial scale.

There is also a limitation regarding the distance to river—elevation weighted layer. Overall, the rivers map layer used to create the DREW indicator in this study covers all 23 major waterways covering the entire study area. Using this layer to create the DREW indicator assumes that each waterway will be equally hazardous in the event of a flood in this region. Whilst indeed being modulated by the elevation layer, this is problematic because not all 23 of these waterways at the same elevation will flood to the same extent or in the same manner during a flood event; nor will they be equally hazardous. As aforementioned, the Hawkesbury and Nepean rivers are the primary rivers downstream of the major Warragamba Dam in the HNC. This means they are subject to additional floodwaters in a flood event that spill from the dam upstream and are consequently two of the most flood-prone and hazardous rivers in the catchment. Therefore, the equal weighting of all equally elevated rivers in the DREW indicator is a limitation of this study. A potential solution to this issue that future research could explore would be to create a flow multiplier for each river in the catchment to modulate the level of hazard of each river.

Finally, future research will combine this FHI with flood-exposure and vulnerability indices, creating a total flood-risk index. This combination process is crucial, because the utility of this FHI could be strengthened by combining it with two other risk components (exposure and vulnerability) and creating a holistic assessment of flood risk.

## 5. Conclusions

This research aimed to create a flood hazard index (FHI) using a case study for the Hawkesbury-Nepean catchment in New South Wales, Australia. This FHI was created as a proxy assessment using three indicators: maximum 3-day precipitation (M3DP), distance to river—elevation weighted (DREW), and soil moisture (SM), each incorporating their own respective element of novelty.

Overall, the developed FHI identified higher flood hazard risk areas located within the HN Valley area in the eastern region of the catchment area. This study is validated in part by the high focus that is already placed on this area by stakeholders and authorities due to the easily flooded choke points and ‘bathtub’ effect that this specific region is widely known for. Thus, the identification of this area as the highest flood hazard risk is a positive and validating result.

Specifically, this high flood hazard risk occurred as a result of the high river density in combination with the extreme rainfall totals recorded, as the M3DP indicator illustrated a general trend of increasing precipitation towards the eastern boundary and decreasing to the west of the catchment. This trend was reflected in the final FHI. The DREW layer was highly influential on and strongly correlated with the FHI, as expected. As this flood-risk assessment was focused on river flooding (as well as pluvial flooding), the river paths were broadly identified as the highest flood hazard risk in a proxy assessment where flood modelling data were unavailable at such large spatial scale. The SM was highly variable across the catchment and had a weak correlation with the FHI. The HN Valley floodplain area recorded relatively lower SM values that contrasted with the broader trend of higher risk values here, leading to a lower correlation overall.

In summary, this research offers a viable proof-of-concept for proxy flood risk assessments (FRAs) over a large-scale area using multi-criteria decision-making (MCDM) techniques. The developed index-based methodology is replicable, scalable, and not resource-intensive, making it a viable alternative to the modelling-based FRAs that are predominantly applied on small spatial scales. Ultimately, floods are worsening in magnitude, frequency, and socioeconomic impact due to the impacts of anthropogenic climate change, rapid urban development on floodplains, and intensifying climate drivers. Thus, this study has the potential to assist relevant decision-makers with developing a proactive approach to flood risk management, contributing to increased resilience of at-risk communities.

## Figures and Tables

**Table 1:** Data collection and indicator metadata.

Indicator	Data Used	Source	Original Resolution	Year
Maximum 3-Day Precipitation	GSMaP rainfall precipitation data	Space-based Weather and Climate Extremes Monitoring (SWCEM)	0.1 deg	2021
Distance to River—Elevation Weighted	Custom NSW Rivers layer via BoM	BoM Geofabric	50 m	2020
Soil Moisture	https://awo.bom.gov.au (accessed on 1 May 2022)	BoM	0.05 deg	2021

**Table 2:** Risk class assignments and their corresponding index values.

Risk Class	Very Low	Low	Moderate	Severe	Extreme
**Risk Value**	0 ≤ 0.2	0.2 ≤ 0.4	0.4 ≤ 0.6	0.6 ≤ 0.8	0.8 ≤ 1

**Table 3:** Relative areas and percentages of each risk category across the Hawkesbury-Nepean catchment.

Flood Risk Category	Percentage of Total	Area (Square Kilometres)
**Very Low **(**0 ≤ 0.2**)	0	0
**Low** (**0.2 ≤ 0.4**)	0.005	1
**Moderate** (**0.4 ≤ 0.6**)	16.4	3561
**Severe** (**0.6 ≤ 0.8**)	60.9	13,232
**Extreme** (**0.8 ≤ 1**)	22.7	4941
**Total**	**100**	**21,735**

**Figure A1:**
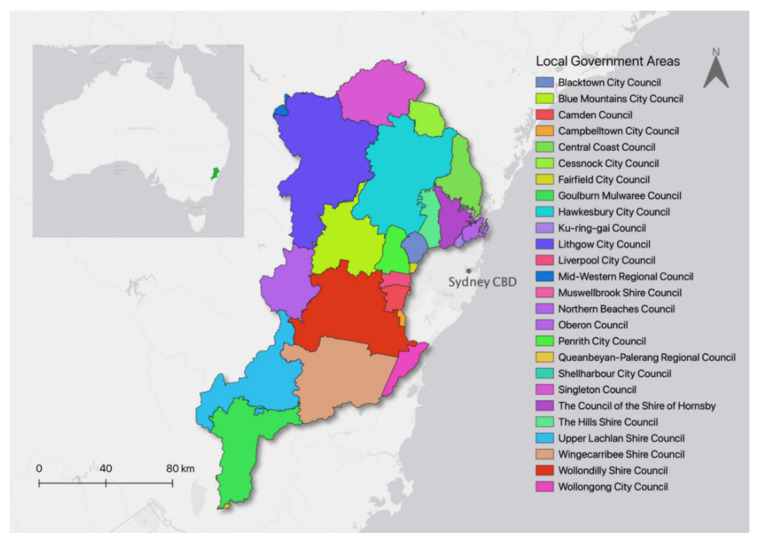
Map of the Hawkesbury-Nepean catchment study area, showing Local Government Area boundaries within the Hawkesbury-Nepean catchment (as of 2022).

**Figure A4:**
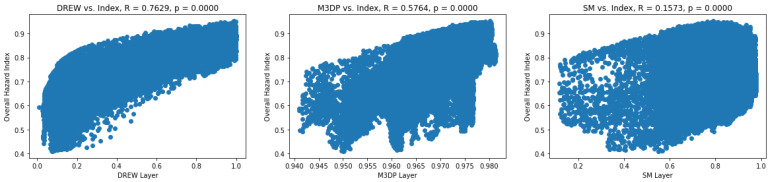
Linear correlation plots between each indicator and the final flood hazard index.

## Data Availability

Not applicable. This study did not report any data supporting reported results.

## Appendix C

**Table A1:** Fuzzy Membership midpoints and spread values.

*Fuzzy* Midpoint and Spread Values	Midpoint	Spread	*Fuzzy Membership* Class
Maximum 3-Day Precipitation (Event Standardising)	281.31 (mm)	5	*Fuzzy Large*
Maximum 3-Day Precipitation (Historical Standardising)	27.5 (mm)	1.5	*Fuzzy Large*
Distance to River—Elevation Weighted	0.15 (normalised value)	2	*Fuzzy Small*
Soil Moisture (Event Standardising)	0.501 (50.1%)	5	*Fuzzy Large*
Soil Moisture (Historical Standardising)	0.3665 (36.65%)	5	*Fuzzy Large*
